# A Perspective on Mature Gratitude as a Way of Coping With COVID-19

**DOI:** 10.3389/fpsyg.2021.632911

**Published:** 2021-03-22

**Authors:** Lilian Jans-Beken

**Affiliations:** The Thriving Human Science Center, Venray, Netherlands

**Keywords:** corona, clinical psychology, positive psychology, character strengths, virtues

## Abstract

**Aim and Methods:**

This perspective presents evidence of mature gratitude as a way of coping with the threats and boundaries of coronavirus disease 2019 (COVID-19). This narrative, non-systematic review will be based on studies from the COVID-19 period in association with more general literature on the characteristics of mature gratitude related to good mental health.

**Results:**

The results from the literature suggest that a confrontation with our existential vulnerability during a pandemic is not only a crisis but also an opportunity to view our lives in a different way. Mature gratitude, as proposed in this perspective, can help us in coping with the threats and boundaries that are part of our lives due to the COVID-19 pandemic. This time of crisis gives us the opportunity to self-reflect on our current life and plans for the future and to reframe them through a positive lens which can encourage individuals to actively strengthen their psychological resilience and coping skills.

**Conclusion:**

Cultivating an attitude of mature gratitude through actions of kindness, expressing being thankful for life and God, and enjoying all the small things in life helps in coping with the current threats of COVID-19 and building lifelong resilience for the future. Knowledge about these associations can help psychologists, counselors, and coaches to support people who experience psychological issues due to the current pandemic and all crises to come.

## Introduction

As the coronavirus disease 2019 (COVID-19) pandemic continues, we all must adjust to a threatening world that was already been scourged by conflict, natural disasters due to climate change, and other serious adversities. The severe acute respiratory syndrome coronavirus 2 (SARS-CoV-2) forces us to physically distance us from others and abstain from important social behavior causing loneliness, anxiety, and depression. We live under lockdown and are strongly advised to refrain from larger gatherings and unnecessary traveling. This leads to disruptive agitation in the population of an individualistic and hedonistic society where immediate need fulfillment now must be postponed for the sake of collective health. Furthermore, there is serious economic damage whereby many people have lost their jobs and face poverty and homelessness. Above all, there is an existential fear that lingers in our daily life now that COVID-19 is threatening mostly the lives of the vulnerable and old, but also young and healthy people are at risk of becoming seriously ill.

These times show the necessity for positive psychology 2.0 (PP 2.0), the successor of positive psychology 1.0 (PP 1.0). Whereas PP 1.0 focuses on the pursuit of happiness (Seligman and Csikszentmihalyi, 2000), it becomes clear that we cannot avoid or ignore unpleasant issues like suffering and human weaknesses, something that was already known in suffering societies across the world before the COVID-19 pandemic. Our mental health is not immune to adverse effects, and we need a way to cope with these disruptive issues acknowledging that suffering is part of living. PP 2.0 posits that life is a struggle in a difficult and dangerous world. The only way to achieve sustainable well-being is to embrace and transform suffering and human weaknesses into our advantage for personal growth, happiness, and success. This can be achieved through learning how to make the best use of the dynamic and dialectic interplay between positive and negative life experiences in each context (Wong, 2019; Wong et al., in press).

### Aim and Scope

The aim of this perspective is to present evidence of mature gratitude as a way of coping with the threats and boundaries due to the COVID-19 pandemic. This manuscript is a narrative review. The purpose of a narrative review is to describe a topic of interest. They have no specified search strategy, are not systematic and do not follow specified protocols (Ferrari, 2015). For this narrative review, studies were included from the COVID-19 period in association with more general literature on the characteristics of mature gratitude such as dispositional gratitude, existential gratitude, state gratitude, spirituality, and religion. Relevant articles were found through database search or references in given articles. Knowledge about these associations can help psychologists, counselors, and coaches to support people who experience psychological issues due to the current pandemic.

### Mature Gratitude

**FIGURE 1:**
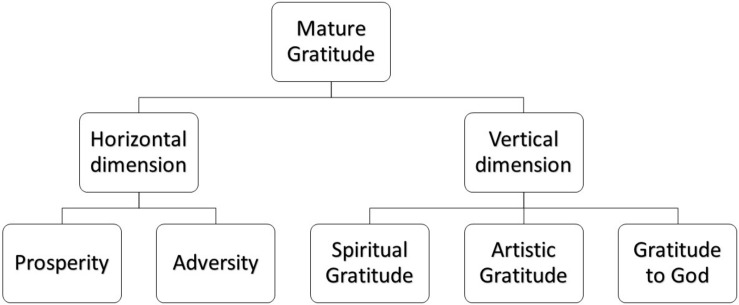
Conceptual model of mature gratitude.

One of the ways to learn to cope with positive and negative life experiences is mature gratitude; a concept associated with positive psychology 2.0 (see Figure 1). This concept arose over time as more and more became known about gratitude and its many facets. In the beginning, trait gratitude was defined as a wider life orientation towards noticing and being grateful for the positive in the world (Wood et al., 2010). A definition of gratitude that includes more facets of life is proposed by Jans-Beken (2018): “Trait gratitude is viewed as a general tendency to recognize small to large benefits, to experience sufficiency, and to acknowledge anything in the world, both human and non-human, with grateful emotion and expression of this emotion which promotes personal well-being and the well-being of others.” (Jans-Beken, 2018, pp. 10–11). Although not explicitly, this definition of an attitude of gratitude already includes two dimensions: a horizontal and a vertical dimension.

The horizontal immanent dimension includes gratitude for prosperity and adversity that is conceived consciously and within earthly borders. The horizontal gratitude is directed at materialistic and naturalistic objects, expected and unexpected events, and the people with whom we interact. Being grateful for the good things in life is the easier part (McCullough et al., 2002). One of the most common gratitude interventions is the Three Good Things exercise which focusses solely on the good and consists of writing down three things that went well that day along with their causes and consequences (Seligman et al., 2005). This intervention is often used in experiments and the results showed that inducing gratitude with the Three Good Things exercise is associated with less psychopathological symptoms and more happiness (Gander et al., 2012; Lomas et al., 2014; Sexton and Adair, 2019). Being grateful for broken objects, disheartening events, and people that are annoying or hurtful, is a more difficult part of gratitude. In times of adversity and uncertainty such as a pandemic, people feel powerless and they lose their sense of control over their life and their faith. If they realize that everything they have and counted on, may be taken away, it becomes hard to take it all for granted. When people become aware of their mortal limitations, this awareness will enhance their sense of gratitude for life. People can gain the most from a grateful perspective on life during a crisis (Emmons, 2013; Frias et al., 2011). In a study by Jans-Beken and Wong (2019) the results showed that gratitude for good and adverse aspects – existential gratitude (Jans-Beken and Wong, 2019) – predicted better well-being in people with symptoms of PTSD, while gratitude for only good aspects – trait gratitude – was not (Jans-Beken and Wong, 2019). This shows that it is necessary for good mental health to accept and transform frustration, powerlessness, and hurt that one experiences into growth and thriving.

The vertical dimension is the experience of gratitude for phenomena that cannot be precisely and mentally located in space and time. Illustrations of this vertical gratitude are cosmic gratitude (Roberts, 2014). Gratitude to God, non-directed existential gratitude (Lacewing, 2016) or spiritual gratitude that can be elicited by, for example, gratitude for ancestors or spirits, but also cultural expressions such as music or art, or an awareness of being part of something big. There are many definitions of spirituality and religiousness where spirituality is considered a personal experiential belief, such as belief in a higher power, or having a sense of belonging with others or the universe. Religiousness includes these personal beliefs, but it also incorporates organizational or institutional beliefs and practices such as church membership and attendance, and commitment to the beliefs system of an organized religion (Zinnbauer et al., 1997).

Spiritual or religious people have a stronger tendency to experience gratitude than do less spiritual or religious individuals (Emmons and Kneezel, 2005). Findings on spirituality and gratitude showed that self-reported spirituality and spiritual behaviors such as prayer and meditation, increased a sense of gratitude. Spirituality brings awareness to an individual’s feelings of gratitude (Lambert et al., 2009; Olson et al., 2018). Thanks giving to God is one of the most basic religious expressions and is one of the most common themes of people’s prayers and descriptions of their religious lives. Being religious might facilitate gratitude in two ways. First, it might amplify the perception of benefits during trying times, and second, by transforming negative experiences by adding spiritual or religious meaning to the event. Even in situations that are distressing, finding meaning in some way can strengthen someone’s sense of gratitude (Frankl, 1959; Krause and Hayward, 2013; Rosmarin et al., 2015). The question arises if non-theist can feel grateful in the first place. Lacewing (2016) posits that a “psychologically rich and satisfying account of appropriate non-directed existential gratitude is available to the non-theist” (Lacewing, 2016, p. 14). Thus, feeling grateful does not necessarily require the vertical dimension but it will deepen and intensify the experience of it. Mature gratitude might play a vital role in preventing people from depression, anger, and anxiety because of suffering, by teaching people a better and adaptive way to embrace their hardship (Jans-Beken and Wong, 2019).

## Mature gratitude and COVID-19

### Threats

In 2020, every country on the Earth faced various threats from the SARS-CoV-2. First, there is the physical threat from the virus itself. Although everyone can get infected by the SARS-CoV-2, especially vulnerable and older people seem to be susceptible to become life-threateningly ill due to COVID-19. Many of us fear losing our elder parents and other elder family members. Some of the patients end up in the intensive care, and when they survive, residual complaints invalidate the survivors for a long time and perhaps even for life (Bij de Vaate et al., 2020). Second, there is an economic threat. For almost all countries in the world, the unemployment rate has risen dramatically (Bureau of Labor Statistics and U.S. Department of Labor, 2020; Eurostat, 2020; Statista, 2020). For most of the people who were able to hold their job or business, income and revenue, respectively, dropped. Many governments provided social safety nets for entrepreneurs and companies to prevent them from going bankrupt, but although this can prevent some in going out of business, there still will be companies that will cease to exist after 2020. Many people have lost the possibility to provide for themselves and their family, increasing poverty and homelessness around the globe (Community Solutions, 2020; Slicker, 2020; Sumner et al., 2020).

Both the physical and economical threat can cause the experience of existential fear; people must cope with the fear of losing a safe home and not being able to provide food or care for their children, and there is a realistic possibility of becoming ill or even die. This existential threat is new for many of us, and it may lead to severe psychological difficulties (Blustein and Guarino, 2020; Son et al., 2020). A review of the early evidence regarding mental health and COVID-19 suggests that anxiety, depression, and self-reported stress are subsyndromal mental health issues following the COVID-19 pandemic (González-Sanguino et al., 2020; Peteet, 2020; Rajkumar, 2020).

Studies from 2020 showed that state and trait gratitude was associated with better mental health. Grateful participants reported less anxiety and depression and better subjective well-being during the COVID-19 pandemic (Bono et al., 2020; Butler and Jaffe, 2020). State gratitude – which is defined as the emotion of gratitude (Fredrickson, 2004) – increased from before to during the pandemic for students from lower SES families, whereas the opposite was observed for students from higher SES families (Bono et al., 2020). Nurses working under immense pressure during the COVID-19 pandemic mentioned in a qualitative study that their gratitude grew for the support from colleagues, relatives, friends, and all sectors of society. Most nurses said that they would continue working and living with gratitude in the future. The respondents who were able to self-reflect on their own values and mortality during the hard times found positive forces to grow in a psychological way (Sun et al., 2020). This is in agreement with the scarcity heuristic explanation which proposes that when individuals are reminded of or confronted with death in a personal manner, they appreciate their own life more (King et al., 2009). Microsoft was one of the companies that immediately introduced working from home and coupled this with a study including gratitude. They asked their employees to reflect daily on their previous day. Data from 4,641 nightly reflection diaries showed that 47% of the employees experienced a positive impact of grateful reflection and 35% reported an increase of feelings of control during these uncertain times (Butler and Jaffe, 2020). Gratitude seems to contribute to mental well-being, also during a crisis such as a pandemic.

Some studies were conducted regarding spirituality and religion in association with mental health during the COVID-19 period. One study from Spain reported higher levels of spiritual well-being as a strong predictor for reduced symptoms of depression, anxiety, and post-traumatic stress disorder (PTSD) during this period (González-Sanguino et al., 2020). In a sample of American Orthodox Jews, direct exposure to COVID-19 was correlated with higher religiosity; positive religious coping, intrinsic religiosity, and trust in God strongly correlated with less stress and more positive impact, while negative religious coping and mistrust in God correlated with the inverse (Pirutinsky et al., 2020). A sample of Iranian adults reported a mediating role of spiritual health on the association between the adverse effects of pervasive anxiety on positive future attitudes and quality of relationship with the family (Kamran and Fazlollah, 2020). A study with American youth found that those who are not religious reported worsening of their mental health during COVID-19 compared to those who consider themselves religious. The adolescents who turned to their faith and those whose religious beliefs helped them through difficult situations reported to have their faith strengthened during COVID-19 (Kang et al., 2020).

A mixed-method study including gratitude and spirituality among women reported that women who considered themselves spiritual but not religious reported that during the pandemic, a certain feeling of being grateful for a connection with God gave them hope. Other women reported that they were grateful for little things in their lives and the opportunity to slow down and spend more time doing joyful activities. Some women explained how they maintained their gratitude, for example, by keeping a gratitude journal and by downward social comparison thinking the situation could be worse (Roberto et al., 2020). These findings highlight that, for some, faith may promote resilience and mental health, especially during crises.

### Boundaries

The first strategy against the spread of the SARS-CoV-2 that many governments issued was the lockdown. The strictness of the lockdowns varied from a strong advice to stay home and work remotely if possible to being prohibited to leave home including a ban on buying tobacco and alcohol (Wasserman and Moynihan, 2020). As the virus spreads by droplets and aerosols that cross a certain distance while talking/laughing/singing/sneezing/coughing, the experts advised to keep a physical distance from other people, which they wrongfully called “social distancing.” However, this physical distance of at least 1 m prohibits people to engage in important social touching behaviors such as shaking hands for trust and comforting others by hugging (Dolcos et al., 2012; Forsell and Åström, 2012). This lack of closeness and affection can lead to psychological distress in healthy people and can deteriorate the mental health of people already suffering from psychological illnesses (Venkatesh and Edirappuli, 2020).

Another problem in maintaining limiting strategies to prevent the spread of a deadly virus to protect the population is the individualistic and hedonistic nature of many societies in 2020. Strategies such as the lockdown are derived from communally oriented East Asian cultures. The way that culturally relevant concepts of rights and freedoms underpin COVID-19 restrictions in individually oriented countries appears to be troublesome. We see people in the streets demonstrating against the restricting policies. People are standing up against the new boundaries because they cannot immediately satisfy their needs to go out or go on a vacation due to the lockdowns and limitations in travelling. They claim that their human rights are being violated and that governments use this pandemic to restrict people’s freedom of movement. They cannot believe that the strategies are there to protect public health and that sticking to the rules contributes to the greater good (Bolsover, n.d.). This anger, based on lingering fear and hopelessness, is also a serious threat to one’s psychological health (Trnka and Lorencova, 2020).

Gratitude was overwhelmingly expressed during the lockdowns across the world. People came to their doorsteps or balconies to clap and make noise to express their gratitude for the effort of frontline staff. A study by Mead et al. (2020) was based on the GENIAL model, which is characterized by a life-course biopsychosocial approach that places individual well-being within the dynamic interplay of individual, social, communal, and environmental ecosystems. They included physical activity, tragic optimism, trait gratitude, social support, and nature connection in their study of mental well-being during the COVID-19 pandemic. According to this study, gratitude was positively associated with mental well-being, along with tragic optimism during the lockdown (Mead et al., 2020). In a qualitative study of patients and carers, the lockdown was mentioned to be a benefit for which one could be grateful. Patients expressed to be grateful for the continuation of their medical treatment despite the pandemic, and carers felt grateful for being able to be at home 24/7 (Bryan et al., 2020). Fear, anger, and hopelessness were the most frequent traumatic emotional responses during the first stage of outbreak of the COVID-19 pandemic (Trnka and Lorencova, 2020). To deal with these traumatic emotions, gratitude, among others, was associated with more adaptive and prosocial responses to the pandemic, realizing that one is not alone on this world but part of a larger whole (Syropoulos and Markowitz, 2020). Thus, gratitude could potentially help regulate the negative impact that a lockdown might have on a person’s mental well-being and their social relationships.

Regarding the concepts of spirituality, and religion, few studies associated with COVID-19, lockdown, and social distancing were available. A Brazilian adult sample mentioned spirituality and religion to be important contributions to the relief of suffering due to the physical restrictions by having an influence on mental health outcomes such as lesser worrying, sadness, and fear (Lucchetti et al., 2020). An issue with religion during the COVID-19 period was the ban of large gatherings of people including live religious services. For many faithful people, attending live services is an important part of their lives and it preserves their mental health (VanderWeele, 2020). However, alternative initiatives to the live religious services were implemented to maintain a sense of belonging of the religious community, which appeared to be able to provide spiritual comfort, religious care, and engagement with the religious community during the pandemic (Frei-Landau, 2020; Ribeiro et al., 2020).

Both gratitude and spirituality were able to contribute to good mental health during the lockdown. In an Italian adult sample, a country severely affected by the COVID-19 pandemic, the virtues in action (VIA) strengths were included to study psychological distress and COVID-19-related self-efficacy. They found that the transcendence strengths—zest, perseverance, gratitude, spirituality, self-regulation, love, and hope—were strongly associated with less psychological distress and more COVID-19-related self-efficacy. Individuals high in transcendence strengths reported higher scores for general mental health; lower scores for psychological distress such as fewer symptoms of depression, anxiety, and stress; and higher scores for self-efficacy in coping with the lockdown situation (Casali et al., 2020).

The hedonic society and the immediate fulfillment of needs shimmered through the study of Bono et al. (2020). They showed that students from higher SES families tended to feel less grateful during the beginning of the pandemic compared to students from lower SES families. This shows that higher SES students, who feel more like having to give up the good life from before the pandemic, tend to use gratitude to cope with the adversity of the pandemic less than their lower-SES peers. An explanation for this can be the gain-loss framing. The pandemic with its threats and boundaries frames the situation for the high-SES students more as a loss than for the low-SES students who might experience losing less or even have a sense of gain (Bono et al., 2020; Fiedler and Hillenbrand, 2020). Thus, regarding the new boundaries due to the COVID-19 pandemic, gratitude and spirituality support coping with handling previous activities differently and contribute to good mental health in such a limiting situation.

## Future Research

Mature gratitude is a new concept associated with positive psychology 2.0 and it fits in existential models such as the dual-systems model by Wong (2012), This model highlights that a life worth living consists of positive and negative conditions and positive and negative outcomes (Wong, 2012). This narrative review shows that mature gratitude with its focus on both positive and negative aspects of life, including spirituality, can have important ramification in coping with crises and trying times such as the COVID-19 pandemic. Mature gratitude is a broader and more comprehensive concept than trait gratitude or Gratitude to God. It does include gratitude to the good and it contains a spiritual dimension, but it also includes gratitude to adversity and suffering. It is a concept that is more applicable in a dangerous and threatening world, more applicable to reality. Future research should investigate mature gratitude in more depth. It is important to know if mature gratitude is a better and stronger predictor for good mental health than the previously frequently used narrower concepts of gratitude. The study of Jans-Beken and Wong (2019) does suggest this better and stronger association and it warrants further investigation into the concept of mature gratitude. Forthcoming studies should include a questionnaire on gratitude with the focus on the good and the bad, such as the Existential Gratitude Scale (Jans-Beken and Wong, 2019) combined with a questionnaire on spirituality such as the Spiritual Coping Questionnaire (Charzyńska, 2014; Jans-Beken, 2019) or the Spiritual Well-Being Scale (Paloutzian et al., 2012). Future research should include longitudinal studies and Experience Sampling Method studies which can help entangle the concept of mature gratitude and its merits.

## Conclusion

The aim of this perspective was to present evidence of mature gratitude as a way of coping with the threats and boundaries due to the COVID-19 pandemic. The narrative review searched for studies from the COVID-19 period in association with more general literature on the characteristics of mature gratitude related to good mental health during a crisis. The above results from the COVID-19 period suggest that a confrontation with our existential vulnerability during a pandemic is not only a crisis but also an opportunity to view our lives in a different way. Mature gratitude as proposed in this perspective can help us in coping with the threats and boundaries that are part of our lives right now due to the COVID-19 pandemic. This time of crisis and fear gives us the opportunity to self-reflect on our current life and plans for the future and to reframe them through a positive lens that can encourage individuals to actively strengthen their psychological resilience and coping skills.

This narrative review is by no means a full account of the literature on mature gratitude and mental health during a crisis. However, many studies that were conducted during the COVID-19 period were included and all of them showed similar results with the literature on gratitude and mental health from before the pandemic. This shows that mature gratitude can still be and perhaps even especially be an important way of coping with the dire circumstances during a crisis such as a pandemic. Cultivating an attitude of mature gratitude through actions of kindness, expressing being thankful for life and God, and enjoying all the small things in life helps in coping with the current threats of COVID-19 and building lifelong resilience for the future (Schiraldi, 2017; Jans-Beken et al., 2019; Kanekar and Sharma, 2020; Mead et al., 2020). Knowledge about these associations can help psychologists, counselors, and coaches to support people who experience psychological issues due to the current pandemic and all crises to come.

## Data Availability Statement

The original contributions presented in the study are included in the article/supplementary material, further inquiries can be directed to the corresponding author/s.

## Author Contributions

The author confirms being the sole contributor of this work and has approved it for publication.

## Conflict of Interest

The author declares that the research was conducted in the absence of any commercial or financial relationships that could be construed as a potential conflict of interest.
